# Customized protective visors enabled by closed loop controlled 4D printing

**DOI:** 10.1038/s41598-022-11629-3

**Published:** 2022-05-09

**Authors:** Qinglei Ji, Xi Vincent Wang, Lihui Wang, Lei Feng

**Affiliations:** 1 Department of Production Engineering, KTH Royal Institute of Technology, 10044 Stockholm, Sweden; 2 Department of Machine Design, KTH Royal Institute of Technology, 10044 Stockholm, Sweden

**Keywords:** Electrical and electronic engineering, Mechanical engineering, Polymers

## Abstract

The COVID-19 pandemic makes protective visors important for protecting people in close contacts. However, the production of visors cannot be increased greatly in a short time, especially at the beginning of the pandemic. The 3D printing community contributed largely in fabricating the visor frames using the rapid and adaptive manufacturing ability. While there are many open source designs of face visors for affordable 3D printers, all these designs fabricate mono-sized frames without considering diverse users’ dimensions. Here, a new method of visor post-processing technology enabled by closed loop controlled 4D printing is proposed. The new process can further deform the printed visor to any customized size for a more comfortable user experience. FEM analysis of the customized visor also shows consistent wearing experience in different circumstances compared with the old visor design. The fabrication precision and time cost of the method is studied experimentally. A case study regarding the reducing, reusing and recycling (3R) of customized visors in classrooms is proposed to enable the customized visors manufactured in a more sustainable way.

## Introduction

Since the breakout of COVID-19, the demand for personal protective equipment is tremendously increased. In different countries, people are suggested or required to wear protective masks or face visors to reduce the spread of virus. However, the storage and production of protective equipment are limited especially at the beginning of the pandemic. Correspondingly, the 3D Printing (3DP) community transferred their rapid prototyping ability to the massive manufacturing of protective equipment like protective face masks^1,2^ and face shields^3^. For example, at KTH Royal Institute of Technology, Sweden, 3D printed protective visors are donated to the local healthcare facilities in the early months of 2020, when the pandemic started in Sweden^4^. Besides the research community, hobbyists from all over the world are also motivated to fabricate the protective components against the crisis. Open source models of these components are shared so that people do not have to redesign the 3DP files from scratch^5^.

As the most commonly 3D printed protective equipment, face shields, or visors, are composed of two simple parts: the 3D printed visor frame and a transparent film^6,7^. The visor frame is designed to be 3D printed directly without using any support material and the transparent film is in the size of the A4 paper and can be easily accessed from any stationery store. The transparent film has holes that are made using a standard paper hole puncher. The holes can be fitted in the designed fixers on the visor frame to form the assembled protective visor.

For the 3DP of visor frames, the most commonly used 3D printers are the Fused Deposition Modeling (FDM) 3D printers, which also hold the largest portion of personal owned 3D printers^8^. FDM 3D printers take fusible polymer filaments and the fused material is then deposited to the building plate to form 3D structures. One of the most commonly used material for FDM 3DP is Polylactic acid (PLA)^9^. PLA is a degradable and bio-compatible material which makes it suitable for skin contact applications^10,11^. It also has good mechanical strength and fatigue performance so that the printed sample can be used for a long time without damage. Furthermore, the material is cost-effective and can be easily 3D printed without any professional training^12^.

However, most of the open source models of the visor frames are mono-sized. They are designed with moderate sizes to fit the majority of people^7,13,14^, which as a result causes some uncomfortable wearing experiences especially for long term usage. Although 3D printing has customizable and adaptive manufacturing ability, it is unpractical to design and printing customized visors with individual sizes for every user. Armijo et al.^15^ use head straps to provide the tightening force for the visor frame. The head straps are attached to the visor frame through manually cut slits and the distances of the slits can be adjusted to change the tightening force. Neijhoft et al.^16^ mention that the 3D printed visor frame can be heated by hot water and then manually adjusted. However, these methods all require users’ careful adjustment, which is not suitable for inexperienced users. Furthermore, no systematic study is performed on how these adjustments can affect the actual wearing tightness.

This paper presents a new manufacturing procedure taking advantage of the thermal induced Shape Memory Effect (SME) of PLA, which belongs to Shape Memory Polymer (SMP)^17,18^. The shape of SMP can change between its permanent shape and the temporary shape. Utilizing this property, two types of 4D printed SMP methods are reported in the literature^19^. The first method^20^ introduces the pre-strain during the printing process by tuning the printing temperature. The printed object has the temporary shape and can then change its shape with temperature stimuli to release the pre-strain after printed. The second method^21^ prints the SMP in its permanent shape. The shape is then deformed to a temporary shape with external force when heated above the glass transition temperature ($T_g$) of the material. The temporary shape can be maintained when the stimulus temperature goes down and the external force is removed. The SMP can then recover to its permanent shape again with temperature stimulus higher than $T_g$. In this study, we will focus on the latter method. By implementing Closed Loop Controlled 4D Printing (CL4DP) over the recovery process, the recovery speed of the SMP can be regulated by changing the stimulus temperature applied on the SMP^22,23^. It is also feasible to terminate the recovery process by decreasing the temperature below $T_g$. Thus, through monitoring the shape of the SMP in real time and regulating the temperature accordingly, arbitrary shape can be achieved using feedback control algorithms.

In this work, the visor frames are 3D printed in a common manner with the mono-size. The frames are then deformed to a temporary shape by heating a local area of the visor. Due to the SME of the material, the shape of the visor will recover to the printed shape when the local area is heated up again. During the recovery process, the local temperature is precisely controlled so that the shape recovery stops at a desired target shape. These post-processing steps enable the visor to achieve any shape to fit the different requirements from users and the new 4D printing method for the visor frame is proved to be more time-efficient than traditional case by case redesign and printing method to enable customization. Following the Finite Element Method (FEM) analysis, the customized visors demonstrate constant wearing experiences for different users compared with traditional mono-sized designs. Furthermore, the already customized visor frames can be reused in the CL4DP process which makes the visors manufacturing process recyclable and sustainable.

## Design and fabrication of the new visor frame

### Redesigned visor frame

**Figure 1:**
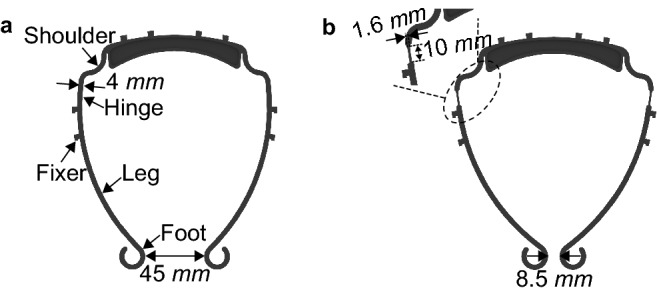
Visor frames. (**a**) Normal visor frame. (**b**) Redesigned visor frame.

As aforementioned, the visor consists of the 3D printed visor frame and the transparent film. To make the visors comfortable to wear for different people, the visor frame sizes are adjusted in the CL4DP post-processing process. The visor frame for print is redesigned to achieve such flexibility. Figure 1 compares the new and the original designs of the visor frame. The distance between the two feet of the redesigned frame is reduced to $$8.5\ $$mm from $$45\ $$mm of the normal size frame by bending the hinges of the visor legs. The length of the hinge area is $$10\ $$mm. For better heating through the entire width of the hinge, its width is also reduced from 4 to $$1.6\ $$mm as shown in Fig. 1b.

### FEM analysis of the heat transfer in the visor frames

**Figure 2:**
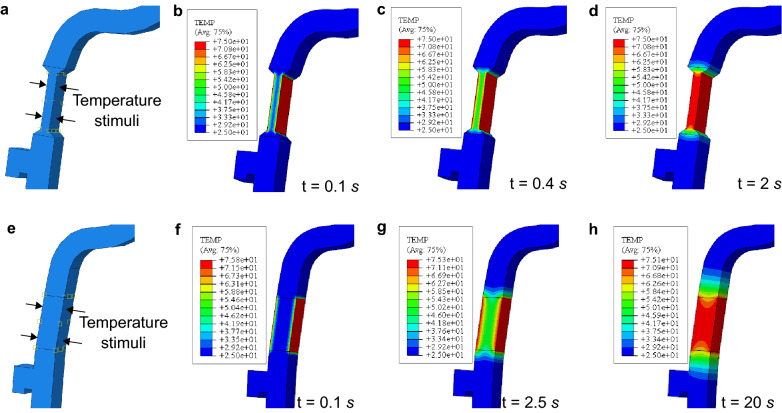
FEM heat transfer analysis in the hinges of the redesigned visor frame (**a**–**d**) and normal visor frame (**e**–**h**) for a temperature stimulus of $75\;{^\circ}$C. (**a**, **d**) The applied temperature stimulus area on the two visor frames respectively. (**b**–**d**) and (**f**–**h**) The temperature distribution of the visor frame at different time stamps. The temperature values in the plots are with units: $degree\ Celsius\ (^\circ$C).

To verify the improvement of heat transfer in the redesigned visor frame under temperature stimuli, FEM studies are performed with the software Abaqus on both the redesigned and normal visor frames and the results are shown in Fig. 2. The thermal conductivity of PLA is set as $0.13\ {\text {W/m}}\ {\text {K}}$ and its specific heat is $1800\ {\text {J/kg}}\ {\text {K}}$. The density of PLA is set as $1240\ {\text {kg/m}}^3$.

Figure 2a shows the distribution area of the temperature stimuli applied for the heat transfer analysis of the redesigned visor frame. The heating area covers the narrowed section of the visor frame. For the heat transfer analysis, the two sides of the narrowed section are heated to $75\;{^\circ}$C and the ambient temperature is $25\;{^\circ}$C. Figure 2b–d show the process of the heat transfer and temperature increasing at different locations of the visor frame. At $$t=0\ $$s, the whole body of the visor frame has the equal temperature as the ambient. At $$t=0.1\ $$s, the temperature stimuli is applied. The two sides of the narrowed section become $75\;{^\circ}$C while the middle area is still $25\;{^\circ}$C as shown in Fig. 2b. Subsequently, the heat penetrates towards the middle part and the temperature starts increasing across the section. Starting from $$t=0.4\ $$s shown in Fig. 2c, every part of the narrowed section has a temperature over the glass transition temperature $T_g = 50\;{^\circ}$C of PLA, meaning that the visor frame can then either be deformed manually or recover under control. Fig. 2d shows the temperature distribution after $$2\ $$s when the visor frame is fully heated. The high temperature area only concentrates in the narrowed section without infecting the other parts.

For comparison, the same FEM analysis is also performed on the normal visor frame by heating the same hinge area as shown in Fig. 2e–h. Figure 2e shows the stimulus area which shares the same position and area as the FEM analysis for the redesigned visor in Fig. 2a. At the heating time of $$0.1\ $$s, a larger portion in the hinge area of the redesigned visor frame has higher temperature. For the normal visor frame, the middle area of the heated hinge reaches the glass transition temperature $T_g$ at $$t=2.5\ $$s, which is more than 6 times longer than heating the redesigned visor frame. The normal visor frame becomes fully heated at $$t=20\ $$s as illustrated in Fig. 2h and is 10 times longer than heating the redesigned visor frame. Furthermore, comparing Fig. 2d,h, we can see that when the two visor frames are fully heated, the heat spreads to a larger area in the normal visor frame. This can cause unwanted deformation in the following CL4DP process. The FEM heat analysis proves that the narrowed section enables the redesigned visor frame to be stimulated at the desired area in a shorter time compared with the normal ones, ensuring the closed loop control of the visor shape with quickly adjusted temperature stimuli.

### 3D printing of the visor frame

The visor frames in this study is 3D printed with an Ultimaker 2+ Connect FDM 3D Printer (Ultimaker B.V.) and the PLA filaments have $$2.85\ $$mm diameter and are provided by 3D Prima. The visor frame structure is optimized for 3D printing and can be directly printed without any supporting material. The time cost for printing one visor frame is around $$6\ $$min and the weight of the 3D printed visor frame is $$14.5\ $$g on average.

## Experimental setup for CL4DP

To customize the dimension of the visor frame, we exploit the SME of the frame material and regulate the precise dimension of the frame through feedback control. The controller requires sensors to measure the actual temperature at the hinge and the dimension of the frame, and an actuator to change the heating temperature applied to the hinge.

### Self-sensed heating unit

**Figure 3:**
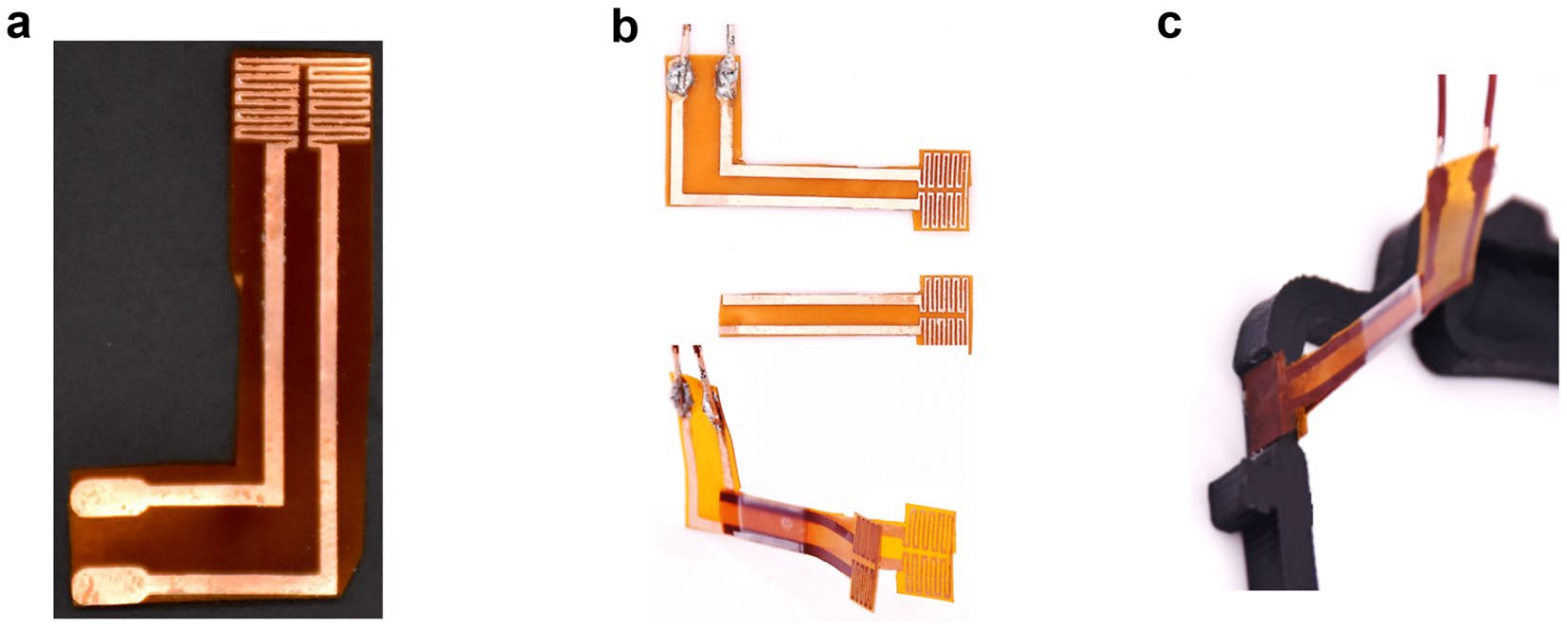
Heating circuit. (**a**) Single sided heating unit. (**b**) Double sided heating unit. (**c**) Double sided heating unit on the visor frame.

Traditional heating devices have separate heating sources and sensors for temperature monitoring which is hard to be applied for the SMP stimulus because the shape of the heated area on the SMP changes and the changing shape can cause unpredictable inaccuracies in heating and sensing. Here a tiny heating unit that can self-sense its temperature to realize fast-response and controllable temperature is applied^22^. The heating circuit is fabricated by the copper etching method. The method uses a single sided Pyralux Flexible copper laminate which is made of a composite Kapton polyimide film with copper foil on one side. The fabricated one-sided heating circuit is shown in Fig. 3a. The thicknesses of the Kapton base and the Copper base are $50\ {\upmu }$m and $30\ {\upmu }$m, respectively. To realize the heating from two sides of the hinges in the visor frame, two one-sided heating circuits are bonded together with tapes as shown in Fig. 3b. The wide exposed copper area of the heating circuits are attached to each other so that one electric voltage input can be used as the power supply for both heating circuits. Details of the etching process can be found in our prior work^22^. Figure 3c shows that the double-sided heating unit is placed on the narrowed section of the visor frame. Unlike the practice in our prior work^22^, there is no need to stick the heating units to the material now as the elastic forces from bending the two flexible heating units are adequate to fix them to the narrowed section of the visor frame.

The copper has resistance coefficient that is linear to temperature^24,25^. The relation can be expressed as:

1 $$\begin{aligned} R(T)=R_{ref}[1+\kappa (T-T_{ref})] \end{aligned}$$

where $R_{ref}$ represents the electric resistance at a reference temperature $T_{ref}$ and $\kappa$ is the temperature coefficient of resistance for the material. Here, $T_{ref} = 20\ ^{\circ }$C and $\kappa = 0.00393$. *R*(*T*) is the resistance at temperature *T*. With Eq. (1), the temperature of the copper circuit can be estimated with the real-time resistance:

2 $$\begin{aligned} T(R)=\frac{1}{\kappa }\left( \frac{R}{R_{ref}}-1\right) +T_{ref} \end{aligned}$$

The electric resistance of the heating circuit is calculated using Ohm’s law with the measured electric voltage and current and then (Eq. 2) is applied to calculate the real time temperature of the heating circuit.

### Image processing for shape feedback

**Figure 4:**
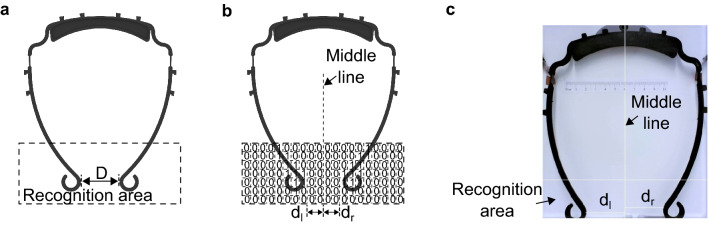
Image processing. (**a**) Image recognition area. (**b**) Recognized visor boundary. (**c**) Camera view with image processing results.

The actual shape of the visor frame during the CL4DP process is monitored by cameras. As illustrated in Fig. 4a, the boundary distance between the two feet of the visor represent the size of the visor, denoted as the visor size *D*. A larger value of *D* indicates a larger size of the customized visor frame, and hence it is more suitable for people with wider forehead. The image sequences from the monitoring camera are transferred from the Blue, Green, Red (*BGR*) color space to the Hue, Saturation, Value (*HSV*) color space for better distinction of different colors. For easier extraction of the visor frame from the images, in our study, the background for taking pictures are set as white while the colors of the visor frames are normally colorful instead of white. In the *HSV* color space, the *H* and *S* values of pure white color are both 0 and the *V* value can vary between 0 and 255. In experiments the pure white color is unachievable, thus the threshold for white color is set as $H = 10, S = 10$ empirically. The *H* and *S* values of every pixel at the recognition area are compared with the threshold. If the values are below the threshold, the pixel is considered as the background area while the other pixels are considered as the visor frame area as illustrated in Fig. 4b. Then the horizontal coordinates of the most right pixel from the visor frame in the left half image area and the most left pixel in the right half image area are used to derive the distance between the visor boundaries to the middle line. The distance of the left foot to the middle line is marked as the left foot distance $d_l$ and the distance of the right foot to the middle line is marked as the right foot distance $d_r$. Thus the visor size *D* is:

3 $$\begin{aligned} D = d_l + d_r \end{aligned}$$

As aforementioned, the redesigned visor frame has an initial size of $$D_0 = 8.5\ $$mm. Thus the initial left and right foot distances are $$d_{l0} = d_{r0} = d_0 = 4.25\ $$mm. Figure 4c shows the image processing result in real time camera view. The distances $d_l$ and $d_r$ are extracted respectively for the later individual control of the two visor legs.

### Cascade control structure

**Figure 5:**
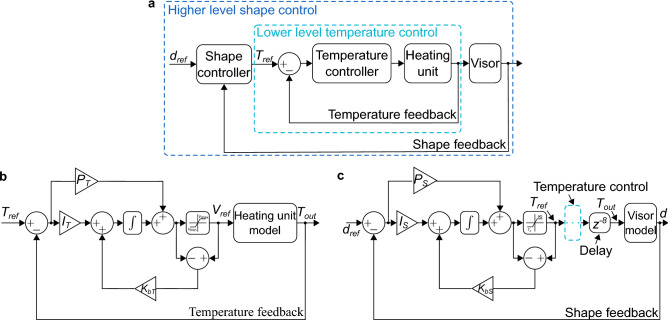
Control structure. (**a**) Cascade control structure. (**b**) Lower level temperature control. (**c**) Higher level shape control.

The shape control of the visor frame has a cascade structure of two levels: the lower level controls the heating temperature by regulating the input electric voltage applied on the heating unit and the higher level controls the left and right foot distances $d_l$ and $d_r$ by regulating the temperature stimuli from the heating unit. The control structure is illustrated in Fig. 5a. The lower level temperature control executes at a sampling time of $$T_s = 0.05\ $$s and the higher level shape control runs at $$10T_s = 0.5\ $$s. Thus for each temperature sent to the temperature controller, it has 10 steps to regulate the actual temperature. The lower level sampling time for the temperature control on the heating unit is around 10 times faster than the time cost $$t = 0.4\ $$s of the temperature penetration throughout the visor frame as explained in Fig. 2, which ensures fast and timely adjustment of temperature stimuli.

For the lower level control, the voltage-temperature response of the heating unit described in Fig. 3 is modeled using experimental data and a PI controller is developed to perform error feedback closed loop control of the heating unit temperature as shown in Fig. 5b. The temperature reference from the output of the higher level controller is considered as the reference for the lower level controller and the temperature feedback is derived from the electric resistance of the heating unit using Eq. (2). Details on the structure and development of the temperature controller will be discussed later.

For the shape control process, the distances of the two legs to the middle line are controlled individually. Thus to obtain a visor frame with a size of $D_{ref}$, the references for the left and right feet are both $d_{ref} = D_{ref}/2$. The shape controller monitors the real time feedback distances $d_l$ and $d_r$ with the image feedbacks and decides the desired temperature reference for achieving the target shape.

For the shape controllers, both model-based and model-free control methods can be applied depending on the requirements. It is demonstrated that high shape precision is required and the fabrication time is not firstly prioritized, a PI controller can be used^22^. On the other hand, if the time-consuming is sensitive while the precision is not the first concern, model-based methods like Dynamic Programming (DP) or model-based Reinforcement Learning (RL) methods can be applied^26^. For simplicity, this study will elaborate the development of a PI controller for the higher level control as shown in Fig. 5c and detailed introductions are presented later.

### Experimental setup

**Figure 6:**
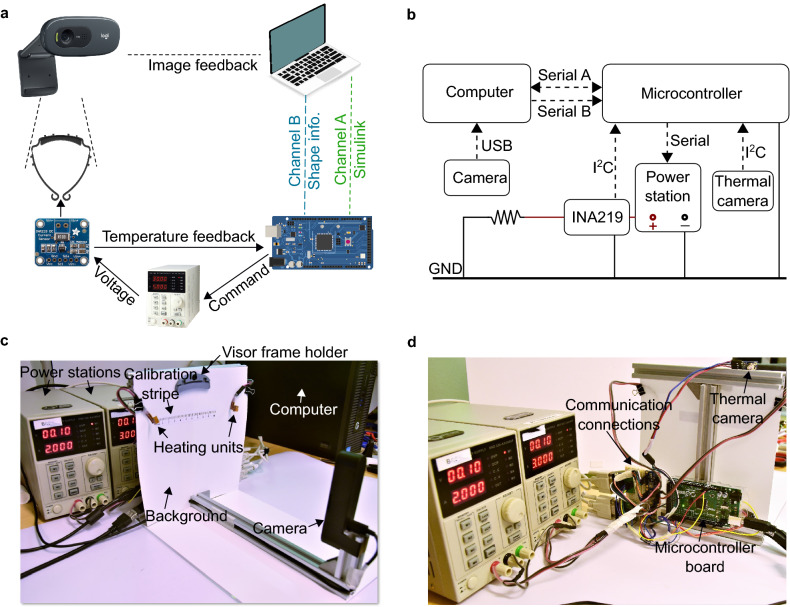
Experiment setup. (**a**) Connections for the experiment setup. (**b**) Schematics of the communications. (**c**) Front view of the experimental setup. (**d**) Back view of the experimental setup.

The diagram of the experimental setup for the visor customization is shown in Fig. 6a. A web camera is used to capture the actual shape of the visor frame and the image sequences are sent to a laptop for image processing and analysis using the Python Language. The signals of the foot distances are sent to the microcontroller board (Arduino Mega 2560 board) for real time measurement and control. On the same computer a MATLAB Simulink Graphical User Interface (GUI) runs and communicates with the microcontroller board to offer the visual monitoring of the system states such as the temperatures of the heating units and the voltages applied on the heating units.

For real time feedback control, the microcontroller board receives the voltage and current signals passing through the heating units from two INA219 High Side DC Current Sensor Breakouts that are connected with the two heating units respectively and then calculates the current resistances of the heating units. The temperature of the heating units are then calculated using Eq. (2). With the temperature and the shape received from the computer, the shape controller outputs the reference temperatures, which are then controlled in the temperature control loop. The output voltages from the temperature controllers are sent to two power stations as commands and the output voltages from the power stations follow the commands and apply the voltages on the heating unit for heating.

Figure 6b shows the connections and communications between devices. The image sequences from the web camera are sent to the computer through USB communication. The computer and the microcontroller board communicate via two serial lines for transmitting the visor frame shape and Simulink signals. The microcontroller board receives the voltage and current information from the INA219 modules through I2C communication and send commands to the power station through serial communication. A thermal camera for temperature calibration purpose is also connected with the microcontroller board via I2C. During the calibration process, the thermal camera is pointed at the heating unit and the temperature readings are used to adjust the $R_{ref}$ value in Eq. (1) until that the estimated temperature matches with the readings from the thermal camera. The thermal camera is then not involved in the latter temperature control process. Note that in real experiments, two INA219 modules and two power stations are used for heating both sides of the visor frame simultaneously and individually but are drawn once for clarity.

Figure 6c,d shows the picture of the experiment setup. Two heating units are placed on the two sides of the background as seen in Fig. 6c. On the background a calibration stripe is used for the calibration of the web camera, which is fixed at a certain distance to the background using aluminum profiles to make the camera view cover the visor frame space. A visor frame holder designed for quick holding and releasing of the visor frame is located in the center of the setup. The two power stations are placed behind the setup as seen in Fig. 6d. The microcontroller board and the connections for communications are all fixed on the back on the setup.

## Model and control

### Model of the visor morphing process

To design the shape morphing controller for the CL4DP of the visor frame, the recovery process of the visor frame is modeled. The constitutive models of the SMP recovery process are deeply investigated in various studies^27–29^. In previous studies, we found that the relation between the strain rate ${\dot{\epsilon }}(t)$ and the strain $\epsilon (t)$ of the SMP recovery process can also be expressed using a data-driven model as^22,26^:

4 $$\begin{aligned} {\dot{\epsilon }}(t)=6.75\times \frac{\epsilon (t)}{R_r}\left( \frac{\epsilon (t)}{R_r}-1\right) ^2(aT+b) \end{aligned}$$

where *t* is the time, *a*, *b*, $R_r$ are the constants to be fitted with experimental data and are only related to the SMP property. The strain is defined as:

5 $$\begin{aligned} \epsilon (t) = \frac{d_m - d(t)}{d_m - d_0} \end{aligned}$$

where $d_0$ is the initial left or right foot distance and $d_m$ is the maximum foot distance during the pre-deformation process. The strain rate ${\dot{\epsilon }}(t)$ can thus be written as:

6 $$\begin{aligned} {\dot{\epsilon }}(t) = \frac{-{\dot{d}}(t)}{d_m - d_0} \end{aligned}$$

The morphing model of the visor frame in Eq. (4) can then be unfolded as:

7 $$\begin{aligned} {\dot{d}}(t)=-6.75\times \frac{d_m - d(t)}{R_r}\left( \frac{d_m - d(t)}{R_r(d_m-d_0)}-1\right) ^2(aT+b) \end{aligned}$$

To acquire the parameters for the visor frame morphing model, $d_m$ is set as a fixed value of $$70\ $$mm, which is larger than the maximum size of the visor frame to be fabricated. Different temperature stimuli are applied as step inputs to the visor frame and the morphing responses are recorded. These response data are then used for the fitting of the model parameters and the detailed fitting process can be found in^22^. The achieved parameters of the visor morphing model are: $a = 0.002$, $b = -0.1$ and $R_r = 0.8$.

### Controller development

The controller development for the cascade control is divided into two stages: design of the lower level temperature controller and design of the higher level morphing controller. Both controllers utilize PI control with anti-windup as illustrated in Fig. 5b,c.

The transfer function model of the heating unit^22^ which represents the temperature of the heating unit and the applied electric voltage is built in MATLAB Simulink and the parameters of the PI temperature controller with anti-windup illustrated in Fig. 5b are then acquired through fine-tuning until no control overshoot is visible and the rise time is as short as possible. The lower voltage limit $V_{\min }$ in the saturation block is used to ensure that there is always current in the heating unit so that the resistance can be measured for calculating the temperature feedback using Eq. (2). The upper voltage limit $V_{\max }$ protects the heating unit from burning out. The parameters for the temperature controller are finally defined as follows: the proportional gain $P_T = 0.3$, the integral gain $I_T = 0.2$, the back-calculation coefficient $K_{bT} = 6$, the lower and upper electric voltage $$V_{\min } = 0.2\ $$V and $$V_{\max } = 0.9\ $$V. 

Similar fine-tuning process using the visor morphing model is performed to acquire the controller for the shape control process as illustrated in Fig. 5c. Before the temperature output from the heating unit is applied to the visor morphing model, a time delay of $$0.4\ $$s are applied to simulate the time delay of the temperature penetrating throughout the visor frame as discussed in Fig. 2. The lower temperature limit in the saturation block is set as the glass transition temperature $T_g$ of the SMP so that the deformation process can start faster when the shape control process starts. The tuning target for the morphing controller is to regulate the visor shape as fast as possible while keeping the static error below $5\%$. Different $P_S$ and $I_S$ values are tested in the simulation model and it is found that larger $P_S$ can increase the rise time while decreasing the static error. On the other hand, larger $I_S$ values decrease the rise time and the static error. Detailed methodologies can be found in our previous report^22^. The final achieved parameters for the shape controller are: the proportional gain $P_S = 2$, the integral gain $I_S = 0.08$, the back-calculation coefficient $K_{bS} = 2$. The higher and lower controllers are then implemented experimentally for controlling the shape morphing of the visor frame to different shapes.

## Results

### Operation steps

Following above discussions, the manufacturing of the customized visors using the CL4DP method is divided into several steps:

1. The redesigned visor frames are 3D printed using an FDM 3D printer as shown in Fig. 7a. After the printing is finished, the visor frames can be customized. Before starting customization, the system is initiated and the temperature of the heating units is calibrated using the thermal camera. The pixel size of the web camera is also calibrated using the calibration stripe on the white background.

**Figure 7:**
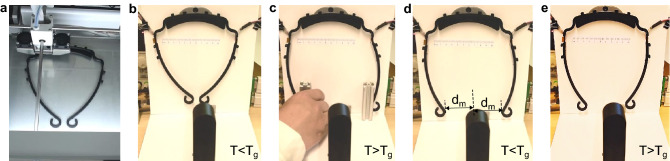
Operation processes for customized visor fabrication. (**a**) 3D print the visor frame. (**b**) Mount the printed visor frame. (**c**) Heat the visor frame and manually deform it to a larger size. (**d**) Cool down the temperature and fix the temporary shape. (**e**) CL4DP process for customized visor shapes.
2. The 3D printed visor frames are placed on the visor frame holder and the double-sided heating units are attached to the hinges of the visor frame on both sides as shown in Fig. 7b. The two heating units are heated up to stimulate the visor frame.
3. After the hinges are fully heated, the legs can be bent outwards manually and fixed with blockers at the foot distance $d_m$ as shown in Fig. 7c, where $d_m$ is the maximum foot distance during the pre-deformation process as marked in Fig. 7d and must be larger than half of the maximum size of the customized visors. This ensures the later CL4DP process to continue. For example, in this study, the maximum size of the fabricated customized visors is $$110\ $$mm. Then $d_m$ should be larger than $$110/2 = 55\ $$mm so that the visor size can change from a larger value to $$55\ $$mm in the CL4DP process. In the process of modeling for the visor morphing, $d_m$ is set as a fixed value of $$70\ $$mm as an example. In the actual control process, $d_m$ can be bent to any value larger than $$55\ $$mm.
4. As illustrated in Fig. 7d, decrease the temperature to cool the visor frame and the enlarged visor frame structure can be maintained when the blockers are removed.
5. Under different size requests, a corresponding reference foot distance is sent to the size control system. The size control system then increases the temperature above $T_g$ to activate the SMP recovery process on both sides and the stimulus temperatures are regulated automatically to control the frame morphing process as demonstrated in Fig. 7e. The final foot distance is controlled to the different references and a visor frame with arbitrary foot distance and wearing tightness is manufactured. The shape control process can be repeated for the same visor frame for multiple times, which enables the recycling usage and will be elaborated later in the case study. More details demonstrating the visor fabrication and customization process are shown in the Supplementary Video S1.

### Demonstration of fabricated visors

**Figure 8:**
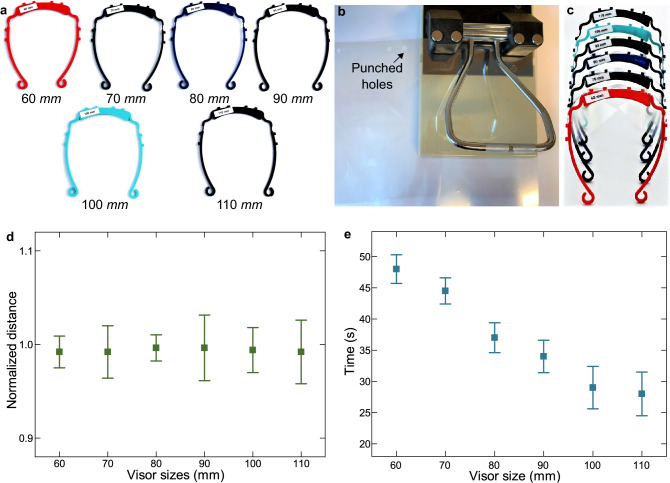
Fabricated visors. (**a**) Demonstration of visor frames with different sizes. The number below each visor frame is *d* which represents the distances between two feet of the visors. (**b**) Punch holes on the transparent films. (**c**) Assembled visors. Performance of the cascade PI controller in terms of (**d**) control accuracy and (**e**) time cost.

Figure 8a shows visor frames with different customized foot distances varying from 60 to $$110\ $$mm fabricated with the developed controllers. Visor frames with different frame colors can also be fabricated using the same system. The visor frame is then assembled with a transparent film for usage. The transparent films with A4 size are punched by a paper puncher at a long edge to have eight holes, as illustrated in Fig. 8b. The transparent films are then assembled with the visor frames to fabricate ready-to-use visors with different sizes as demonstrated in Fig. 8c. The transparent film weighs $$8.7\ $$g and the weight of an assembled visor is $$23.2\ $$g on average.

### Analysis of the manufacturing precision and time cost

The precision and time cost for the CL4DP of visors are then tested with the developed controllers experimentally. For each visor shape, the control tests are performed for 5 sequential times for 3 newly printed visor frames. Fig. 8d plots the controlled foot distance accuracy of the cascade PI controller for different foot distance references. The normalized controlled foot distance distributions are plotted versus the different foot distance references. It can be observed that the controlled distances are all within $5\%$ of the reference values.

Figure 8e shows the distributions of rise time for the same cases. The rise time is defined as the time duration from starting the control till the distance reaches $98\%$ of the reference foot distances. It can be observed that small size visors takes longer control time than bigger sizes as the feet have to move for a longer trajectory. The time cost varies more for customized visors with larger sizes.

### Force analysis of the visor frames

**Figure 9:**
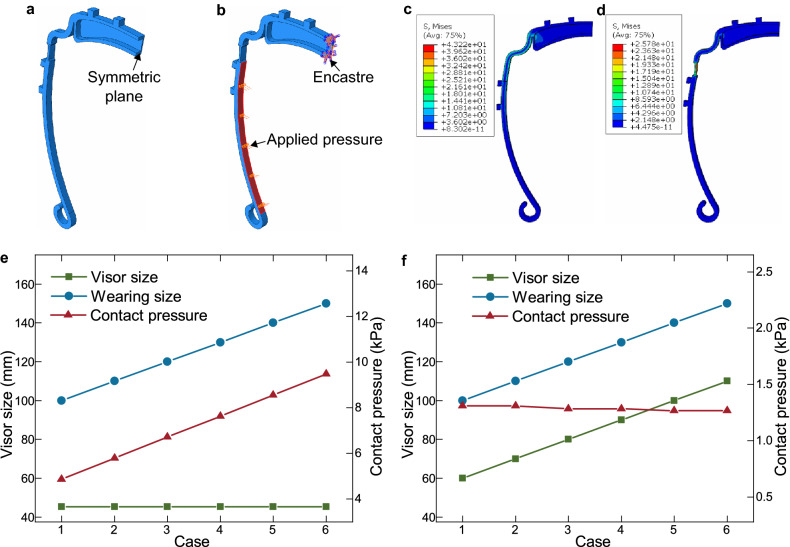
FEM analysis results of two visors. (**a**) Half of the visor frame for FEM analysis. (**b**) Boundary conditions and loading on the visor frame for simulation. (**c**,**d**) Stress distribution on the two visor frames for deforming to foot distance of $$100\ $$mm. (**c**) represents the original visor deformation and (**d**) represents the customized visor deformation with a customized foot distance of $$60\ $$mm. (**e**) Contact pressures from FEM analysis for different wearing sizes of the original visor frame. (**f**) Contact pressures from FEM analysis of the customized visor frame.

FEM studies on the visor frames are performed to study the change of stress distribution of the original visor frame in Fig. 1a and the new designed visor frame in Fig. 1b using the FEM software Abaqus. The contact force of the two visor frame with different human head sizes are also simulated. As the visor frame shape and the forces on the visor frame are symmetric to the middle line as shown in Fig. 4, FEM analysis is performed on half of the visor frame as shown in Fig. 9a. Consequently, an *Encastre* boundary condition is applied on the cross-section of the symmetric plane on the visor. To simplify the simulation, the applied loads onto the visor frame is assumed to be uniform pressure distributed on the two legs of the visor as illustrated in Fig. 9b. The pressure values are considered as the contact pressure between the human head and the visor frame. For the FEM analysis, the material of the visor frame is set as PLA with the Tensile Modulus of $$3.3\ $$GPa and the Compression Modulus of $$2.9\ $$Gpa. The Poisson’s ratio is 0.35. Tetrahedral elements with sizes of $$1\ $$mm are used to mesh the visor frames. Figure 9c,d show the comparison of the stress distribution of the two visor frames with the same final foot distance of $$100\ $$mm. The original visor frame in Fig. 9c deforms from $$45\ $$mm and the customized visor frame in Fig. 9d is deformed from $$60\ $$mm. It can be observed that the shoulder and hinge of the original visor frame undertakes large stress up to $$43.2\ $$MPa while the narrowed part of the customized visor frame takes more deformation and has stresses up to $$25.8\ $$MPa, which is around 2/3 of the original visor frame.

When the visor frames are worn, no matter what the initial sizes are, the visor frames will be bent to a larger size to match the head sizes of the users. The deformed visor size after the visor frames are worn is noted as wearing sizes. We assume six wearing cases where the wearing sizes vary from 80 to $$130\ $$mm with a step of $$10\ $$mm between different cases. The visor frame with the original design is firstly applied with different loads until the foot distances reach the wearing sizes. Fig. 9e shows the contact pressures in the six scenarios for the normal visor frame. The visor sizes remain at $$45\ $$mm while the wearing size increases gradually. Consequently, the contact pressures also increase from $$4.9\ $$kPa per side to $$9.5\ $$kPa, which makes the wearing experience quite different for different people. On the other hand, the CL4DP method fabricates visor frames with sizes $$40\ $$mm smaller than the wearing sizes as shown in Fig. 9f. For example, if the size of the visor after wearing $$100\ $$mm, then the fabricated visor using CL4DP is $$60\ $$mm. As a result, the visor size changes for different scenarios are all around $$40\ $$mm. Thus the contact pressures are also similar in different cases as indicated by the FEM results in Fig. 9f. The contact pressures are around $$1.3\ $$kPa and a very small contact pressure drop can be noticed for larger wearing sizes. The difference of contact pressures are less than $3\%$, which exhibits excellent consistences in wearing experiences with a large variation in wearing sizes.

## Case study: 3R of customized visors in classrooms

The used visors can be easily recycled and reused by cycling the operation steps of CL4DP without the need of reprinting new visor frames, thus reducing the consumption of raw manufacturing materials. During the years of 2020 and 2021 when the COVID-19 pandemic spreads, special safety rules are made for the teaching activities in the educational institutes in many countries. For example, at Department of Machine Design of KTH Royal Institute of Technology, Sweden, students are required to wear visors for on site courses. These courses normally repeats twice or four times a year and visors have to be distributed to the students. To increase the comfort of wearing and reduce the amount of materials used for the preparation of visor frames, we employ the 3R process for the manufacturing of visor frames.

**Figure 10:**
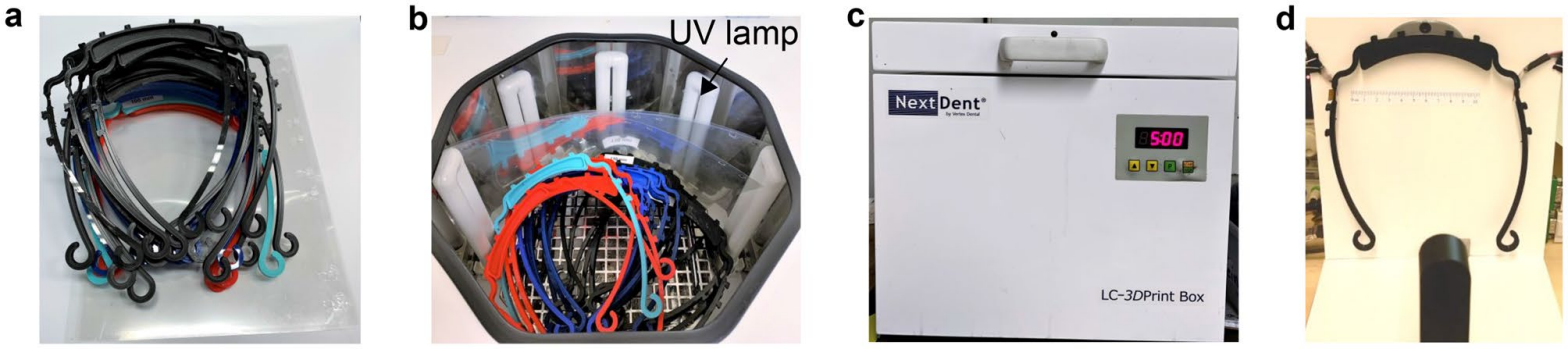
3R process for customized visors. (**a**) Collect used visors and disassemble them to visor frames and transparent films. (**b**) Put the visor frames and transparent films in the UV light box. (**c**) Close the lid of the UV light box and perform UV disinfection for five minutes. (**d**) Repeat the customization process for reuse of the visors.

For the first round of courses, new visor frames are printed and customized following the needs of different students. The exact sizes of customization follow the guidance in Fig. 9f to ensure that constant and comfortable pressure are applied for different students. When the course finishes, the visors are first collected from the users and disassembled to visor frames and transparent films as shown in Fig. 10a. Then a UV light box from Nextdent B.V. is used to disinfect the parts to avoid virus spreading through the visors. The UV light box has 12 UV light bulbs inside the box to generate full light spectrum, which can disinfect the parts effectively and the disinfection process lasts for $$5\ $$min as shown in Fig. 10b,c. When the next round of courses starts, the recycled visor frames can be re-customized following the new students’ needs by repeating the CL4DP process shown in Fig. 10d.

Taking a class with 50 students as an example and assuming that the visors are used for four sequential courses per year, during the two years of implementation, the material usage of PLA are reduced from $50 \times 8 \times 14.5\ {\text {g}} = 5.8\ {\text {kg}}$ to $50 \times 14.5\ {\text {g}} = 0.725\ {\text {kg}}$, which is only $12.5\%$ of the consumption when the visors are not recycled. The cost of PLA is reduced from around $\$145$ to only $\$18$ and the impact to the environment is also decreased. This reduction of consumption can further be increased with time and by applying the method to a larger range of groups.

## Conclusion

This work develops a new visor manufacturing process based on CL4DP in confronting the COVID-19 crisis. A post-processing process is supplemented to the traditional visor frame 3D printing process. The method takes advantage of the SME of the PLA used for 3D printing and is enabled by CL4DP technology. The tightness of the visor frame can be arbitrarily adjusted following users’ requirements.

The experimental setup enabling the above process including both hardware and software is introduced. FEM heat transfer analysis is performed on both normal and redesign visor frames and proves the effective temperature stimuli across the narrowed section. FEM stress analysis is also performed and the redesigned visors demonstrate better and more consistent user experience in different use cases. The data-driven modeling of the heating unit and the visor morphing process are introduced. PI controllers with anti-windup are developed and tested experimentally to study the control precision and time cost for fabricating visors with different sizes. At last, a 3R process applied in university classrooms is also introduced to enable the manufacturing and usage of customized visors in a more sustainable manner.

In the future, the study can be improved in the following aspects: (1) the integration of the manufacturing system can be improved. (2) Automated CL4DP process can be studied to avoid any labor requirements in the whole process. (3) The supply chain issues can be studied when employing the method in a larger scale.

## Supplementary Information

Supplementary Legends.

Supplementary Video S1.
