# Supplementary material for: Customized protective visors enabled by closed loop controlled 4D printing
Source: Sci Rep. 2022 May 9;12:7566. doi: 10.1038/s41598-022-11629-3 (PMC9082988; doi:10.1038/s41598-022-11629-3)
Supplement: Supplementary file 1 — Supplementary Legends. [file 41598_2022_11629_MOESM1_ESM.pdf]

Supplementary Information

"Customized protective visors enabled by closed loop controlled 4D printing"

Qinglei Ji[1,2], Xi Vincent Wang[1], Lihui Wang[1], and Lei Feng[2,*]
[1]Department of Production Engineering, KTH Royal Institute of Technology, Stockholm 10044, Sweden
[2]Department of Machine Design, KTH Royal Institute of Technology, Stockholm 10044, Sweden
[*]lfeng@kth.se

This file includes:
1. Legends of Supplementary Movies S1

1. Legends of Supplementary Video S1

1.1 Supplementary Video S1:

Customized protective visors enabled by closed loop controlled 4D printing

The video demonstrates how the visor fabrication and customization process is performed, including Step 1: Visor frame model design, Step 2: 3D printing of the visor frames, Step 3: Customization of frame shapes, Step 4: Visors ready to use, Step 5: Reduce & Reuse & Recycle.
